# Predictive Factors of Response in HER2-Positive Breast Cancer Treated by Neoadjuvant Therapy

**DOI:** 10.1155/2013/854121

**Published:** 2013-04-29

**Authors:** S. Guiu, M. A. Mouret Reynier, M. Toure, B. Coudert

**Affiliations:** ^1^Medical Oncology, Centre Georges François Leclerc, 21000 Dijon, France; ^2^Medical Oncology, Centre Jean Perrin, 63000 Clermont-Ferrand, France; ^3^Oncology Department, CHU Treichville, BP V3 Abidjan, Cote D'Ivoire

## Abstract

Since 2005, major progresses have been made in the neoadjuvant treatment of HER2-positive breast cancer. Trastuzumab introduction associated with chemotherapy has been the first major step leading to the improvement of the complete pathological response rate and, like in the adjuvant studies, better survivals. Dual HER2 blockade has been the next step and trastuzumab is associated now with other anti-HER2 therapies like lapatinib or pertuzumab, the latter being much more easy to use in combination with chemotherapy. Additional knowledge is necessary to better define within the HER2 tumor subgroup which patients could benefit more from targeted therapies. Different biomarkers have been studied to predict the response after anti-HER2 neoadjuvant therapies but until now none has been validated.

## 1. Introduction

Neoadjuvant chemotherapy in breast cancer treatment is now recognized as a standard care to increase conservative surgery [1, 2]. Its utility is also documented in inflammatory or locally advanced tumors [3]. Long-term results of neoadjuvant chemotherapy are equivalent to those obtained with adjuvant chemotherapy if locoregional treatments are fully applied [1, 2].

In HER2-positive breast cancer, randomized studies with and without anthracyclines have demonstrated the essential role of anti-HER2 therapies in obtaining increased pathological complete response rates and good long-term results.

## 2. Neoadjuvant Anti-HER2 Therapies and Their Impact on the Pathological Complete Response Rate

### 2.1. Trastuzumab

Several randomized trials have evaluated, in the neoadjuvant setting, the role of trastuzumab (Herceptin, Roche laboratory), a recombinant humanized monoclonal antibody that targets HER2 receptor. The first randomized trial in patients with operable noninflammatory disease was stopped early when the pathological complete response (pCR) rate in the trastuzumab group was more than twice as high as that of the control group (65% versus 26%) [4, 5]. The pCR rates in the following studies varied between 26% and 40% in the trastuzumab arms [6–9]. These differences can be explained by various inclusion criteria, different type, and different duration of the regimens. Nonetheless, all the studies showed a higher pCR rate when trastuzumab was combined with chemotherapy compared to chemotherapy alone (Table 1). In the ABCSG-24 study, 536 patients were randomized to receive either 6 cycles of EDC (epirubicin, docetaxel, and capecitabine) or 6 cycles of ED (epirubicin, Docetaxel) [7]. Patients with HER2-positive tumors were also randomized to receive trastuzumab or not. In the 512 eligible patients for efficacy, the pCR rate was significantly higher after neoadjuvant EDC (23.8% versus 15.2%, *P* = 0.036). In the HER2-positive subgroup (*n* = 90), the addition of trastuzumab to the chemotherapy increased the pCR rate (40% versus 26.7%) but this result was not statistically significant (*P* = 0.37). Authors suggested it might be due to the unexpectedly high rate of pCR achieved in the ED/EDC group (the sample size had been calculated to detect a difference in pCR rate of 20% after ED or EDC versus 50% after ED or EDC plus trastuzumab; power = 80%, *P* < 0.05). The lowest pCR rate is reported by Pierga et al. [9]. In this study, trastuzumab was introduced later after anthracyclines and concomitantly with docetaxel while in the other studies, trastuzumab was given upfront for a longer period of time [4, 7, 8].

In the GeparQuattro study, 445 patients with HER2-positive tumors were scheduled to receive 4 cycles of epirubicin plus cyclophosphamide and then were randomly assigned to either 4 cycles of docetaxel or 4 cycles of docetaxel plus capecitabine or 4 cycles of docetaxel followed by 4 cycles of capecitabine [10]. All patients received trastuzumab concomitantly to chemotherapy. Forty percent of patients showed no invasive residual tumor in the breast and had no histologic nodal involvement at surgery (ypT0/is, ypN0). The pCR rates were similar between the 3 groups and the overall breast conservation rate was 63.1% [10].

### 2.2. Lapatinib

Despite improvement of pCR rates, all patients do not equally benefit from trastuzumab therapy. Indeed multiple pathways can contribute to acquired or intrinsic resistance to trastuzumab [21, 22]. Lapatinib (Tyverb, GSK laboratory), a small molecule inhibitor of the tyrosine kinase activity of EGFR and HER2, was approved for use in combination with capecitabine for the treatment of HER2-positive metastatic breast cancer patients who have progressed on trastuzumab [23, 24]. 

Trastuzumab and lapatinib have well-characterized synergistic interaction in HER2-positive breast cancer models [25–27], due to partly nonoverlapping mechanisms of action. Indeed, trastuzumab inhibits ligand-independent HER2 and HER3 signaling and extracellular domain cleavage, triggers antibody-dependent cellular cytotoxicity (ADCC), and involves a reduction of angiogenesis and a decrease of DNA repair [21]. Lapatinib blocks ligand-induced heterodimer signaling and prevents signaling via a frequently expressed truncated version of the HER2 receptor (p95) that could render cells resistant to trastuzumab.

Lapatinib leads also to an accumulation of HER2 at the surface of the cell, enhancing trastuzumab-dependent ADCC [27, 28]. These data are in favor of a dual HER2 blockade. In heavily pretreated patients, the combination of trastuzumab and lapatinib significantly prolonged progression-free and overall survivals as compared with lapatinib alone [29, 30]. 

Table 2 summarizes the randomized neoadjuvant studies with lapatinib versus trastuzumab or lapatinib versus trastuzumab versus lapatinib plus trastuzumab. The pCR rates reported here correspond to the absence of invasive carcinoma both in breast and axilla. 

The direct comparison in the GeparQuinto trial showed that pCR rate with chemotherapy and lapatinib (30%) was significantly lower than that with chemotherapy and trastuzumab (44%) [13]. The results are similar in the randomized phase 2 GEICAM 2006-14 study with a pCR rate in the trastuzumab group twice higher than that in the lapatinib group, with the same chemotherapy regimen as in the GeparQuinto trial [17]. Surprisingly in this study, the breast-conserving surgery rates were similar in the 2 arms. The inferiority of the lapatinib compared to trastuzumab has already been highlighted in the interim analysis of the MA.31/EGF108919 trial (metastatic first line) with a shorter progression-free survival in the lapatinib arm (8.8 versus 11.4 months) [31]. Moreover, the Independent data monitoring committee of the ALTTO study (a phase III four-arm study of adjuvant lapatinib, trastuzumab, their sequence, and their combination) indicated that the lapatinib alone arm was unlikely to meet the prespecified criteria to demonstrate noninferiority to trastuzumab alone with respect to disease-free survival and patients assigned to this arm were offered to discontinue lapatinib and to potentially receive trastuzumab [32].

In the NeoALTTO study, pCR rate was significantly higher in the group given lapatinib and trastuzumab (47%) than in the group given trastuzumab alone (28%) [12]. The results were similar in the nocomparative, randomized phase 2 CHER-LOB study with an anthracycline- and taxane-based chemotherapy [14]. Pathological complete response rates were not significantly different between the lapatinib alone arm and the trastuzumab alone arm in these two studies. In the NSABP B-41 study, although the pCR rate in the dual-blockade arm was 60%, the difference was not statistically significant [15]. It should be noted that only 63% of patients completed the treatment in this dual-blockade arm (essentially due to digestive toxicity), compared to 78% and 68% in the trastuzumab and lapatinib arms, respectively (*P* = 0.01) [15].

### 2.3. Pertuzumab

Pertuzumab (Perjeta, Roche Laboratory) is a humanized monoclonal antibody directed at the dimerization domain of HER2. Pertuzumab and trastuzumab have complementary mechanisms of action due to their distinct binding sites. Pertuzumab inhibits ligand-dependent signaling particularly between HER2 and HER3 which is known to activate a proliferation pathway [33]. Pertuzumab in association with trastuzumab and docetaxel as compared with trastuzumab plus docetaxel significantly prolonged progression-free survival when used as first line in the metastatic setting (18.5 versus 12.4 months, *P* < 0.001) [34]. NeoSphere is a proof-of-concept phase II study with 4 neoadjuvant arms: docetaxel plus trastuzumab (arm A), docetaxel plus trastuzumab plus pertuzumab (arm B), trastuzumab plus pertuzumab without chemotherapy (arm C), and docetaxel plus pertuzumab (arm D, added after a protocol amendment and hence not formally compared to arm A) [18]. Three hundred ninety two patients (of 417 eligible) underwent surgery. Thirty two percent of patients had locally advanced breast cancer and 7% an inflammatory tumor. The pCR rates (defined by no invasive carcinoma in the breast) were 29% (arm A) versus 45.8% (arm B), *P* = 0.0141; 16.8% (arm C) and 24% (arm D). The superiority of the pertuzumab-trastuzumab arm either in first-line metastatic setting or in the neoadjuvant setting leads to the initiation of the ongoing adjuvant trial with pertuzumab (NCT01358877). 

## 3. Breast-Conserving Surgery (BCS) after Neoadjuvant Anti-HER2 Therapies

In a recent meta-analysis, Valachis et al. studied the overall effect of neoadjuvant trastuzumab on BCS [35]. Breast-conserving surgery is scarcely described even in the randomized studies and the number of patients who underwent BCS was available in only four trials [35]. They did not found any difference in terms of BCS between the treatment arms (with or without trastuzumab) (OR: 0.98, 95% CI: 0.80−1.19, *P* = 0.82) [35]. This could partially be explained by the fact that data from only 280 patients was available for analysis. Moreover inclusion criteria varied among studies and one study included T4 tumors [7]. 

In another meta-analysis, Valachis et al. reported the effect of lapatinib versus trastuzumab or the combination (lapatinib plus trastuzumab) versus trastuzumab on the BCS rate [36]. They found no difference in terms of BCS between the treatment arms [36]. Once again, the number of studies and patients is relatively small and can affect the power of the meta-analysis to reveal statistically significant results (five trials and 1442 patients for trastuzumab versus lapatinib; 3 trials and 734 patients for trastuzumab versus combination) [36]. 

Moreover, other criteria can influence the BCS rate and must be taken into account like the tumor size, the presence of in situ carcinoma, the multifocality of the lesion, and the preference of the patient. Unfortunately, these data were not reported in the different studies. 

## 4. Data of Survival after Neoadjuvant Anti-HER2 Therapies 

Most of the studies in this review did not report data of survival. In the NOAH trial, the primary end point was the event-free survival (EFS) [8]. Adjuvant trastuzumab was administered only in patients randomized to receive trastuzumab preoperatively for one year (excepted for 7% of patients). There was a 41% reduction in risk of recurrence, progression, or death with addition of trastuzumab. The 3-year EFS rates were 71% with trastuzumab versus 56% without trastuzumab (HR = 0.59, 95% CI: 0.38–0.90, *P* = 0.013), although 19 (16%) patients with HER2-positive disease assigned to chemotherapy alone crossed over to receive adjuvant trastuzumab [8]. Benefits of trastuzumab were seen in all subgroups tested, including patients with inflammatory disease. The 3-year overall survival rates were 87% with trastuzumab versus 79% without trastuzumab (*P* = 0.114) [8].

Buzdar et al. reported a better disease-free survival (DFS) among patients randomized to chemotherapy plus trastuzumab compared to patients with chemotherapy alone (*P* = 0.041) after a median followup of 36.1 months [5]. DFS was a secondary end point in this study and patients did not receive adjuvant trastuzumab. Among the chemotherapy alone group, the 1-year DFS rate was 94.7% (95% CI: 85.2–100%) and the 3-year DFS rate was 85.3% (95% CI: 67.6-100). There has been no recurrent disease in the patients randomized to chemotherapy plus trastuzumab and the estimated DFS at both 1 and 3 years was 100% (1-year DFS estimate: 95% CI, 85.2–100) [5]. 

## 5. Biomarkers for Response

Many studies reported different biomarkers for the prediction of the pCR after neoadjuvant chemotherapy in HER2-positive breast cancer. Nonetheless, no biomarker is clearly validated so far.

### 5.1. Role of Hormone Receptors (HR) on the pCR Rate

Estrogen-receptor- (ER-) negative tumors are usually associated with a higher pCR rate compared to ER-positive tumors [37–39] including HER2-positive tumors [40]. In the retrospective study of Guarneri et al., the rate of pCR was 15% in patients with HR+/HER2+ tumors versus 29% in patients with HR−/HER2+ tumors (*P* < 0.001), after an anthracycline- and taxane-based therapy without trastuzumab [40].

In neoadjuvant trials with trastuzumab or others anti-HER2 therapies, similar results are described. The pCR rates in HR+ and HR− subgroups are summarized in Table 3.

It has been suggested that ER/HER2 crosstalk may be implicated in the escape from HER2-directed therapy. Wang et al. described an increase of ER and its downstream products in 80% of ER+/HER2+ cell lines after a treatment with trastuzumab and lapatinib. Moreover, acquisition of resistance after trastuzumab and lapatinib could require the activation of the ER pathway via Bcl2 family members [41]. 

### 5.2. Activation of PI3K Pathway

HER2 overexpression leads to an activation of multiple signaling pathways including the PI3K/Akt pathway that result in the modification of cell growth, differentiation, and survival. In a recent study of Dave et al, after neoadjuvant trastuzumab 18.2% (4/22) of patients with low expression of PTEN (IHC) or PI3K mutations achieved pCR whereas 66.7% (6/9) of patients without low PTEN or PI3K mutations achieved pCR (*P* = 0.015) [42]. These findings are consistent with the results published by Berns et al. [43] who used a large-scale unbiased RNA interference screen and identified PTEN as the only modulator of trastuzumab sensitivity, and patients with activation of PI3K pathway had worse clinical outcome. Nagata et al. showed that patients with PTEN-deficient tumors had a poorer response to trastuzumab-containing therapy [44]. With lapatinib, the opposite effect was observed, with 92.3% (12/13) of patients with low PTEN achieving pCR compared with 41.2% (7/17) of patients with normal PTEN [42]. PI3K mutations were not associated with response or resistance to lapatinib in this study (*P* = 0.007) [42]. Hence, low PTEN expression could select patients with tumors resistant to trastuzumab but sensitive to lapatinib and could be used as a biomarker if the results are confirmed by several large phase 3 studies. 

These results are discussed in other studies: in a retrospective study of 129 patients with HER2+ tumors including 26 cases treated with neoadjuvant trastuzumab and 48 metastatic breast cancer receiving a combination of taxane and trastuzumab, any relationship was found between PTEN loss and/or PI3K mutations and response to trastuzumab [45]. A lower clinical benefit rate and a lower overall response rate were observed by Wang et al. on 57 patients treated by lapatinib and capecitabine for metastatic HER2+ breast tumors with PI3K pathway activation (PI3K mutation and/or PTEN expression loss) [46].

### 5.3. p95HER2

Another potential mechanism of resistance to trastuzumab is the accumulation of truncated forms of the HER2 receptor that lack the extracellular trastuzumab-binding domain. Amino terminally truncated carboxyl terminal fragments of HER2, collectively known as p95HER2, are frequently found in HER2+ breast cancer cell lines and tumors [47]. The truncated p95HER2 receptor retains a highly functional HER2 kinase domain where lapatinib can bind. Indeed, lapatinib monotherapy or in combination with capecitabine seems to be equally effective in patients with p95HER2-positive and p95HER2-negative breast tumors [48].

In preclinical models and in 46 metastatic patients treated with trastuzumab, the expression of p95HER2 was correlated with the lack of response to trastuzumab [49]. Loibl et al. reported the pCR (noninvasive residuals in breast and lymph nodes) and resistance rates in 153 patients included in the GeparQuattro study and then received neoadjuvant trastuzumab according to p95 expression in immunohistochemistry [50]. The pCR rate in the p95-positive tumors (10% cutoff) was 58.2% versus 32.6% in the p95-negative group (*P* = 0.009). The resistance rate in the p95-positive group was 25.8% versus 48.7% in the p95-negative group (*P* = 0.014) [50]. Results were similar when the cutoff was set at 20% and 30% as well as for clinical response. In this translational study, p95 expression indicates response to neoadjuvant trastuzumab but not resistance, in contrast to what was expected [50].

In the CHER-LOB trial the expression of p95HER2 was determined by immunohistochemical assay and was found to be present in 30.7% of cases. However, pCR rates were not different for p95HER2-positive and p95HER2-negative tumors in any treatment group [14]. 

### 5.4. HER2 Serum Level

The extracellular domain of the HER2 protein can be cleaved from the surface by metalloproteases and detected in the peripheral blood as serum HER2 (sHER2). The remaining cleaved receptor is constitutively activated, suggesting that the presence of sHER2 also reflects a biological process leading to a more aggressive tumor behavior [47]. Trastuzumab inhibits shedding of HER2 [51]. sHER2 can be measured by enzyme-linked immunosorbent assay (ELISA). In the GeparQuattro trial, Witzel et al. found a significant positive association between pCR and elevated sHER2 levels (above 15 ng/mL, *P* = 0.045) and a decrease of sHER2 levels during neoadjuvant therapy (*P* = 0.02) which was also significant in multivariate analysis (OR = 3.29, 95% CI: 1.001–10.89, *P* = 0.049) [52]. In 210 patients of the GeparQuinto trial (52% received trastuzumab and 48% lapatinib), higher prechemotherapy sHER2 levels were associated with higher pCR rates (OR = 1.8, 95% CI: 1.02–3.2, *P* = 0.043) [53]. Moreover, a decline of sHER2 levels (>20%) during neoadjuvant therapy (after 4 cycles) was a predictor of pCR in the lapatinib-treated group only (OR = 11.7, 95% CI 1.03–110, *P* = 0.031) [53].

Nonetheless, the biological relevance of elevated sHER2 is still unknown and other studies have reported a limited predictive utility of baseline sHER2 [54].

### 5.5. Other Biomarkers

Before neoadjuvant chemotherapy with trastuzumab, circulating miR-210 levels were significantly higher in 11 patients who had residual disease than in 18 patients who achieved a pCR (*P* = 0.0359) [55]. 

Some data suggested that the fragment C *γ* receptor (Fc*γ*R) polymorphism has an effect on ADCC, which is one of the mechanisms of action of the trastuzumab. Tamura et al. observed in the tumors of 15 patients that the Ff*γ*R2A-131 H/H polymorphism predicts the pathological response to trastuzumab-based neoadjuvant chemotherapy in early-stage breast cancer [56].

Esteva et al. suggested that in 45 patients with HER2+ tumors who received concomitant trastuzumab and paclitaxel followed by FEC, a lower expression of genes involved with CD40 signaling was associated with a greater risk of residual disease [57].

In the NOAH trial, in 171 patients with available biopsies, negative progesterone receptor and c-Myc amplification were associated with higher pCR rates after addition of trastuzumab compared to chemotherapy alone. Overexpression of membranous IGF1R was associated with higher likelihood of residual disease after trastuzumab-based chemotherapy [58].

In the exploratory study presented by Holmes et al. at the 2011 ASCO meeting, 100 patients with stage II/III HER2+ breast cancer were randomized to trastuzumab, lapatinib, or trastuzumab plus lapatinib [16]. Before and after the anti-HER2 therapy all patients had core needle biopsies for tissue microarray, stem cell analysis, and reverse-phase protein microarrays, measuring 60 different phosphoprotein/posttranslationally modified protein signaling and gene expression analysis endpoints [16]. Molecular profiles suggested that nonresponders use autophagy and stem-cell-related pathways to evade therapy, while responders have disruption of HER2-HER3 linkages and known downstream regulators of growth and transcription [16].

### 5.6. pCR Prediction by Positron Emission Tomography

In HER2-negative and HER2-positive tumors, the standard uptake value (SUV) decrease, studied with positron emission tomography (PET), is a strong predictor of pCR after only one course of chemotherapy [59]. Other studies are confirmative of these data [60–62]. However PET baseline characteristics and metabolic response to neoadjuvant chemotherapy are highly dependent on the histologic type of breast cancer (i.e., luminal versus HER2-positive versus triple-negative tumors) [63]. By multivariate analysis, ΔSUV was found as the only independent predictive factor of pCR in HER2 subtype. A decrease of SUV over 75% (ΔSUV < −75%) had a high odds ratio (OR) of 6.31 (95% confidence interval = 1.10–39.16; *P* < 0.03). To identify an optimal threshold for the prediction of the pathological response, receiver operating characteristics (ROC) analysis gave an optimal cut-off value of −70% for ΔSUV. At this cut-off value, ΔSUV had a sensitivity of 89% while the specificity was 87%. From these results, an SUV decrease greater than 70% allowed for the early identification of the highly responsive tumors, which will be completely eradicated by trastuzumab-based neoadjuvant therapy, with an accuracy of 76% [63]. These data are currently challenged in a prospective study (AVATAXHER Roche no. ML 22229, Eudract no. 2009-013410-26, NCT01142778) aiming at confirming the role of early PET to differentiate the excellent responders to HER2 neoadjuvant trastuzumab-based therapy from the less responders who are then randomized to additional neoadjuvant bevacizumab [64]. This study is the first one to adjust therapeutic choice according to initial PET results. If successful, this type of strategy could be used to lighten or to reinforce future neoadjuvant treatments. 

## 6. Conclusion

Trastuzumab (Herceptin) has been a breakthrough in the treatment of HER2-positive breast cancer. Trastuzumab combined with chemotherapy has improved response rates, pathological complete response, progression free survival and survival in the neoadjuvant setting of these cancers. With the emergence of new anti HER2 therapies like lapatinib (Tyverb), pertuzumab (Tarjeta) and TDM1 (Kacyla), dual HER2 blockade, still associated with chemotherapy, has proven to be superior. In order to better individualize targeted therapies in specific tumor subgroups, additional knowledge is necessary. Different biomarkers have been studied to predict the response to anti-HER2 neoadjuvant therapies but until now, except the HER2 positivity, none has been validated. PET oriented strategy could be used to separate the most responsive tumors from the less responsive ones. Further studies are necessary to define robust, reliable, and reproducible biomarkers.

## Figures and Tables

**Table 1:** Randomized trials with neoadjuvant trastuzumab.

Study (ref.)	No. of patients	Clinical stage	Neoadjuvant chemotherapy	pCR rate (%)	cCR (%)	Breast-conserving surgery rate (%)
Buzdar et al. [4, 5], phase III	42	II–IIIA	P (4 c) → FEC (4 c)	*Trastuzumab: 65% *No trastuzumab: 26%	Trastuzumab: 87% No trastuzumab: 47%	Trastuzumab: 53% No trastuzumab: 56.5%
H2269s [6], phase II	30	II-III	Doc + carboplatin (4 c)	^†^Trastuzumab: 40% ^†^No trastuzumab: 7.1%	NR	NR
ABCSG-24 [7], phase III	90	T1–4 (except T4d), any N	ED ± Cap (6 c)	^ #^Trastuzumab: 40% ^#^No trastuzumab: 26.7%	NR	Trastuzumab: 69% No trastuzumab: 79%
NOAH [8], phase III	235	T3N1, T4, or any T N2-3	Doxo + P (3 c) → P (4c) → CMF (3 c)	*Trastuzumab: 38% *No trastuzumab: 19%	Trastuzumab: 87% No trastuzumab: 74%	^‡^Trastuzumab: 23% ^‡^No trastuzumab: 13%
REMAGUS 2 [9], phase II	120	II-III	EC (4 c) → Doc (4 c)	*Trastuzumab: 26% *No trastuzumab: 19%	Trastuzumab: 34% No trastuzumab: 22%	Trastuzumab: 47% No trastuzumab: 47%

pCR: pathologic complete response; cCR: clinical complete response; NR: not reported; P: paclitaxel; FEC: 5-fluorouracil + epirubicin + cyclophosphamide; Doc: docetaxel; ED: epirubicin + docetaxel; Cap: capecitabine; Doxo: doxorubicine; CMF: cyclophosphamide + methotrexate + 5-fluoro-uracile; EC: epirubicin + cyclophosphamide.

pCR: *no invasive residual tumor in breast and axilla; ^†^no invasive residual tumor in breast only; ^#^definition not reported.

^‡^[11].

**Table 2:** Randomized trials with neoadjuvant lapatinib versus trastuzumab or lapatinib versus trastuzumab versus lapatinib plus trastuzumab.

Study (ref.)	No. of pts	Clinical stage	Neoadjuvant chemotherapy	Neoadjuvant anti-HER2 therapy	pCR rate (%)	cCR (%)	Breast-conserving surgery rate (%)
NeoALTTO [12], phase III	455	T > 2 cm, any N	Weekly P (12 w)	(i) Trastuzumab 4 → 2 mg/kg/w (ii) Lapatinib 1500 mg/d (iii) Lapatinib 1000 mg/d + trastuzumab 2 mg/kg/w	28% 20% 47%	NR	39% 43% 41%
GeparQuinto [13], phase III	615	cT3/4a–d or HR− or cT2cN+ HR+ or cT1pN_SLN+_ HR+	EC (4 c) → Doc (4 c)	(i) Trastuzumab 8 → 6 mg/kg/3 w (ii) Lapatinib 1250 mg/d (adjustment to 1000 mg/d)	44% 30%	33% 28.5%	64% 59%
CHER-LOB [14], phase II	121	II–IIIA	Weekly P (12 w) → FEC (4 c)	(i) Trastuzumab 4 → 2 mg/kg/w (ii) Lapatinib 1500 mg/d (iii) Lapatinib 1000 mg/d + trastuzumab 2 mg/kg/w	25% 26% 47%	NR	67% 58% 69%
NSABP B-41 [15], phase III	464	T > 2 cm, any N	AC (4 c) → P (D1, 8, 15, 28; 4 c)	(i) Trastuzumab 4 → 2 mg/kg/w (ii) Lapatinib: 1250 mg/d (iii) Lapatinib 750 mg/d + trastuzumab 2 mg/kg/w	49% 47% 60%	82% 70% 77%	54% 46% 50%
Holmes et al. [16], phase II	100	II-III	FEC75 (4 c) → weekly P (12 w)	(i) Trastuzumab 4 → 2 mg/kg/w (ii) Lapatinib 1250 mg/d (iii) Lapatinib 750 mg/d + trastuzumab 2 mg/kg	54% 45% 74%	NR	NR
GEICAM 2006-14 [17], phase II	99	T > 2 cm or N+ any T	EC (4 c) → Doc (4 c)	(i) Trastuzumab: 8 → 6 mg/kg/3 w (ii) Lapatinib: 1250 mg/d	48% 24%	NR	58% 58%

pCR: pathological complete response; cCR: clinical complete response; NR: not reported; HR: hormonal receptors; EC: Epirubicin + Cyclophosphamide; Doc: Docetaxel; FEC: 5fluoro-uracile-epirubicine-cyclophosphamide; AC: adriamycin-cyclophosphamide.

**Table 3:** Rates of pCR according to HR status.

Study (ref.)	Neoadjuvant regimen	pCR rate HR+	pCR rate HR−	*P*
NeoSphere [18]	(i) Docetaxel + trastuzumab—(arm A) (ii) Docetaxel + trastuzumab + pertuzumab (arm B) (iii) Trastuzumab + pertuzumab (arm C) (iv) Docetaxel + pertuzumab (arm D)	20% 26% 5.9% 17.4%	36.8% 63.2% 27.3% 30%	NR
Neo-ALTTO [12]	(i) Weekly P + trastuzumab (ii) Weekly P + lapatinib (iii) Weekly P + trastuzumab + lapatinib	22.7% 16.1% 41.6%	36.5% 33.7% 61.3%	NR
CHER-LOB [14, 19]	(i) CT + trastuzumab (ii) CT + lapatinib (iii) CT + trastuzumab + lapatinib	25% 22.7% 35.7%	26.6% 35.7% 56.2%	NR
Buzdar et al. [4]	(i) CT + trastuzumab (ii) CT alone	61.5% 27.2%	70% 25%	NR
NOAH [20]*	(i) CT + trastuzumab (ii) CT alone	18% 17%	48% 22%	0.51 0.002
REMAGUS 02 [9]	(i) CT + trastuzumab (ii) CT alone	20.5% 20.5%	32% 19%	NR
NSABP B-41 [15]	(i) CT + trastuzumab (ii) CT + lapatinib (iii) CT + trastuzumab + lapatinib	46.7% 48% 55.6%	65.5% 60.6% 73%	NR

*ER positive versus ER negative, pCR: pathological complete response; HR: hormone receptors; P: paclitaxel; CT: chemotherapy; NR: not reported; ER: estrogen receptors.
